# A Comprehensive Analysis of the Role of hnRNP A1 Function and Dysfunction in the Pathogenesis of Neurodegenerative Disease

**DOI:** 10.3389/fmolb.2021.659610

**Published:** 2021-04-12

**Authors:** Joseph P. Clarke, Patricia A. Thibault, Hannah E. Salapa, Michael C. Levin

**Affiliations:** ^1^Department of Health Sciences, College of Medicine, University of Saskatchewan, Saskatoon, SK, Canada; ^2^Office of the Saskatchewan Multiple Sclerosis Clinical Research Chair, University of Saskatchewan, Saskatoon, SK, Canada; ^3^Division of Neurology, Department of Medicine, University of Saskatchewan, Saskatoon, SK, Canada; ^4^Department of Anatomy, Physiology and Pharmacology, University of Saskatchewan, Saskatoon, SK, Canada

**Keywords:** RNA binding protein, hnRNP A1, post-translational modifications, RNA metabolism, neurodegenerative diseases

## Abstract

Heterogeneous nuclear ribonucleoprotein A1 (hnRNP A1) is a member of the hnRNP family of conserved proteins that is involved in RNA transcription, pre-mRNA splicing, mRNA transport, protein translation, microRNA processing, telomere maintenance and the regulation of transcription factor activity. HnRNP A1 is ubiquitously, yet differentially, expressed in many cell types, and due to post-translational modifications, can vary in its molecular function. While a plethora of knowledge is known about the function and dysfunction of hnRNP A1 in diseases other than neurodegenerative disease (e.g., cancer), numerous studies in amyotrophic lateral sclerosis, frontotemporal lobar degeneration, multiple sclerosis, spinal muscular atrophy, Alzheimer’s disease, and Huntington’s disease have found that the dysregulation of hnRNP A1 may contribute to disease pathogenesis. How hnRNP A1 mechanistically contributes to these diseases, and whether mutations and/or altered post-translational modifications contribute to pathogenesis, however, is currently under investigation. The aim of this comprehensive review is to first describe the background of hnRNP A1, including its structure, biological functions in RNA metabolism and the post-translational modifications known to modify its function. With this knowledge, the review then describes the influence of hnRNP A1 in neurodegenerative disease, and how its dysfunction may contribute the pathogenesis.

## Introduction

The molecular mechanisms that define neurodegenerative diseases are as diverse and complex as the diseases themselves. While core mechanisms are shared by all cells (e.g., RNA metabolism, protein translation, ATP production, cytoskeletal growth), the downstream effects of changes in these mechanisms vary across neurodegenerative diseases. Pathological perturbations in cellular pathways that may be a major component of one neurodegenerative disease may not underlie others. Despite mechanistic heterogeneity, however, several commonalities have been identified to help understand neurodegenerative disease pathophysiology. Prominent observations shared amongst neurodegenerative diseases, including Alzheimer’s, Parkinson’s, and Huntington’s are the toxic accumulation of misfolded, insoluble protein inclusions within the cytoplasm or nucleus (Chung et al., 2018). While the toxic accumulation of proteins has been observed for decades in these neurologic diseases, current research has further expanded upon this phenomenon to include new toxic protein targets in several neurodegenerative conditions. Interestingly, several of these protein targets have been identified as RNA binding proteins (RBPs), which contribute significantly to RNA metabolism (i.e., RNA transcription, pre-mRNA splicing, mRNA transport, translation, sequestration, and degradation). This has led to the hypothesis that neurodegenerative disease is a result of altered RNA metabolism and its downstream consequences [reviewed in Liu et al. (2017)].

The heterogeneous nuclear ribonucleoproteins (hnRNPs) have become an intriguing target in neurodegenerative disease research as they are RBPs and several of them are mechanistically linked to pathophysiology (Kashima et al., 2007; Chen et al., 2008; Kim et al., 2013; Cho et al., 2014; Dombert et al., 2014; Moursy et al., 2014; Borreca et al., 2016). HnRNPs are the most abundantly expressed RBPs in mammalian cells, constituting of approximately 20 major types of proteins, and play a major role in all facets of RNA metabolism, especially RNA splicing (Beyer et al., 1977; Pinol-Roma et al., 1988; Dreyfuss et al., 1993). Additionally, within subgroups, hnRNPs share similar structural properties, such as the hnRNP(A/B) subfamily, which includes hnRNP A1, A2/B1, A3 and A0 (hnRNP A1 is focused upon and discussed in this review), however, between subgroups structural properties can differ significantly, which is reviewed in detail (Geuens et al., 2016). For example, hnRNP A1 and hnRNP A2/B1 utilize two RNA recognition motifs (RRMs) for RNA binding, both found in their N-termini, while the hnRNP family member hnRNP K utilizes three K homology (KH) domains, two found in the N-terminus and one in the C-terminus (Geuens et al., 2016). Additional structural differences occur amongst the hnRNPs, including re-organization of similar domains within the N- and C- termini, and/or the inclusion of different domains for added functions such as DNA/RNA binding and protein interaction (Geuens et al., 2016). Together, the intricate structural properties of each hnRNP dictate their individual cellular targets and functions.

In this review we describe hnRNP A1, initially focusing upon its structure and what is known about its cellular functionality. Throughout, we interweave the effects of post-translational modifications (PTMs) on hnRNP A1 and how they have been shown to regulate hnRNP A1 function. We then describe the involvement of hnRNP A1 in the pathogenesis of different neurodegenerative diseases, with an aim to highlight the many open questions about the role, regulation, and effects of PTMs on hnRNP A1 in disease pathogenesis.

## HnRNP A1: Structural Characterization and Role in Liquid-Liquid Phase Separation

### Genetics, Molecular Structure, and Functional Regions of hnRNP A1

**FIGURE 1:**
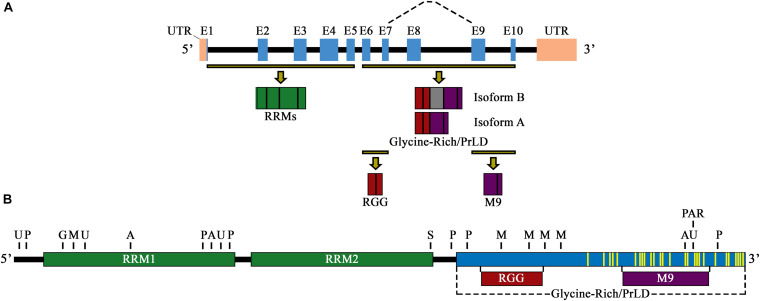
**(A)** Schematic illustration of the primary pre-mRNA transcript and alternative mRNA splicing of hnRNP A1. Exons are labeled E1–E10 and are highlighted by blue boxes. Black boxes indicate introns, and pink boxes are UTRs. Highlighted exon fractions (yellow-black lines) that form the N-terminal and C-terminal regions of hnRNP A1 are illustrated in green (RRM1 and RRM2) and a combination of red (RGG domain) and purple (M9 sequence), forming the glycine-rich/PrLD domain, respectively. The dashed line represents the main spliced region of hnRNP A1, with inclusion of E8 (gray box) constituting isoform B, and its exclusion constituting isoform A. **(B)** Schematic illustration of the primary protein structure and functional domains of hnRNP A1 isoform A. RRM1 and RRM1 are signified by green boxes, and the glycine-rich/PrLD domain by a blue box. Highlighted protein fractions that form the RGG domain (red box) and the M9 sequence (purple box) are noted and are in the glycine-rich/PrLD domain relative to their placement underneath. PTM sites of ubiquitination (U), phosphorylation (P), O-GlcNAcylation (G), acetylation (A), sumoylation (S), methylation (M), and PARylation (PAR) are noted above the protein illustration (selectively representative; refer to Table 1 for full list of PTM locations). Yellow lines delineate mutations sites found in hnRNP A1 that have been published and are associated with the neurodegenerative diseases ALS/FTLD and MS.

The hnRNP A1 gene (Ensembl symbol ENSG00000135486) is located on chromosome 12q13.13 and has two main characterized isoforms, hnRNP A1-A (isoform A, 320 amino acids, NM_002136.4→NP_002127.1) and hnRNP A1-B (isoform B, 372 amino acids, NM_031157.4→NP_112420.1), with hnRNP A1-A being 20 times more abundantly expressed, especially in neuronal cells (Figure 1A; Kamma et al., 1995). Like other members of the hnRNP(A/B) subfamily, both hnRNP A1 isoforms are similarly divided into two main structural portion: an N-terminal portion that includes two RRMs, and a C-terminal portion known as the prion-like domain (PrLD), which mediates cellular compartmentalization, protein-protein interaction, and RNA-binding (Figure 1B; He and Smith, 2009). Protein crystallization of the N-terminal portion of hnRNP A1 shows the two RRMs arranged in tandem and composed of four β-sheets adjoined by two α-helices, in a structure composed of β1α1β2β3α2β4 (Shamoo et al., 1997). The β-sheet surfaces, opposite the α-helices, primarily interact with RNA via amino acid stretches containing predominantly aromatic residues, with two conserved phenylalanine (Phe) residues, Phe17 and Phe59, being necessary for RNA binding (Merrill et al., 1988; Jones et al., 2001). The RRMs are each approximately 90 amino acids long and participate in general and specific RNA and messenger RNA (mRNA) binding through a docking platform, rather than a crevice, allowing for a high degree of RNA accessibility (Gorlach et al., 1992). Although both RRMs share a high degree of homology (∼35% identical and ∼60% similar), they are not redundant and operate as functionally distinct domains, able to bind to distinct RNAs and mRNAs (Casas-Finet et al., 1993; Mayeda et al., 1998). Their sequence binding specificity, however, is similar, as hnRNP A1 prefers to bind to AU-rich elements (AREs), or UAGGGA(U)-motifs present in the 3′-untranslated regions (3′-UTR) of messenger RNA transcripts (Hamilton et al., 1993; Burd and Dreyfuss, 1994; Shamoo et al., 1997).

### Biochemical and Biological Characterization of Functional Regions of hnRNP A1

Biochemical characterization of the C-terminus of hnRNP A1 has shown that hnRNP A1 includes an Arginine-Glycine-Glycine domain (RGG domain), a glycine-rich, PrLD, and a 38-amino acid, nuclear localization/export sequence (NLS/NES), referred to as M9 (Figure 1B). The RGG domain of hnRNP A1 has been shown to influence RNA binding specificity and strength, G-quadruplex DNA binding and unfolding, and in mediating protein-protein interactions (Nadler et al., 1991; Cartegni et al., 1996; Fisette et al., 2010; Hudson et al., 2014). The PrLD is a low complexity domain that promotes both the liquid-liquid phase separation (LLPS) and the self-associative fibrillization propensity of hnRNP A1, as studies on the hnRNP(A/B) subfamily homolog hnRNP A2/B1 show this domain contains a steric-zipper motif that can potentially forms the spine of an amyloid fibril through two self-complementary β-sheets (Kim et al., 2013; Molliex et al., 2015; Xiang et al., 2015; Franzmann and Alberti, 2019).

The role of the hnRNP A1 PrLD in LLPS is important because it processes metastable de-mixing of proteins and/or RNA thought to be mediated by transient and diverse multivalent interactions (Molliex et al., 2015; Shin et al., 2017; Bracha et al., 2018; Gomes and Shorter, 2019). Briefly, LLPS enables the formation of biologically relevant intracellular assemblies of macromolecules in non-membrane-bound cellular compartments, referred to as membraneless organelles, such as stress granules (SGs), processing bodies, nuclear paraspeckles, Cajal bodies, nuclear stress bodies, the centrosome, and the nucleolus (Hyman et al., 2014; Molliex et al., 2015; Zaslavsky et al., 2018; Gomes and Shorter, 2019). Thought to be most important to neurodegeneration, SGs have been shown to assemble via LLPS in response to environmental stressors, and sequester and protect cytoplasmic mRNAs, proteins, and stalled translation complexes (Protter and Parker, 2016; Youn et al., 2019). However, it must be noted that these functions have been challenged recently utilizing high power, single molecule live-cell imaging techniques (Wilbertz et al., 2019). SGs also indirectly promote the specific translation of stress pathway proteins that are involved in mitigating the cell stress effect by limiting global cellular translation (Protter and Parker, 2016; Youn et al., 2019). Once the stressor has been removed, SGs disassemble and release their sequestered components. Current research suggests that alterations in RBP LLPS, thought to occur due to mutations that affect protein-protein interactions, can influence the formation and stability of SGs, and can promote neurodegeneration (Molliex et al., 2015; Protter and Parker, 2016; Gomes and Shorter, 2019; Youn et al., 2019). An evolving theory with hnRNP A1 is that it can inadvertently self-associate, either due to mutation, an adverse PTMs or due to a lack of RNA binding, and may indirectly induce LLPS of other PrLD-containing proteins, many of which are found in SGs (e.g., Ras-GTPase-Activating Protein SH3-Domain-Binding Protein, G3BP1; TAR-DNA binding protein-43, TDP-43; Fused In Sarcoma, FUS; and T-Cell-Restricted Intracellular Antigen-1, TIA1), thereby catalyzing SG assemblies independent of cell stress signaling (Hyman et al., 2014; Molliex et al., 2015; Protter and Parker, 2016; Markmiller et al., 2018; Youn et al., 2019). Recent studies give support to this idea, as PrLD mutations in hnRNP A1 can lead to its aggregation, and subfamily homolog hnRNP A2/B1 LLPS and aggregation are prevented *in vitro* with RNA binding (Kim et al., 2013; Lee and Levin, 2014; Molliex et al., 2015; Ryan et al., 2020).

Finally, the M9 sequence mediates the nuclear import and export of hnRNP A1, however, the amino acid sequences that mediate both functions are indistinguishable within M9 (Michael et al., 1995). Studies have shown that hnRNP A1 nucleocytoplasmic import is mediated by a cytoplasmic interaction with the transport receptors Transportin 1 and 2 (TNPO1/2), both β-karyopherin family proteins, resulting in hnRNP A1 import from the cytoplasm to the nucleus (Rebane et al., 2004; Allemand et al., 2005). This is an active process that requires the utilization of the RanGTP/RanGDP gradient at the nucleopore, and the nucleopore complex (NPC) (Rebane et al., 2004). In addition to mediating hnRNP A1 nucleocytoplasmic transport, TNPO1 has also been shown recently to be a molecular chaperone of hnRNP A1 and has been demonstrated to prevent and reverse hnRNP A1 aggregation (Guo et al., 2018). Nuclear export, however, is not as well understood, as its export is not mediated by exportin 1 transport and is thought to be controlled by PTMs of hnRNP A1 (Lichtenstein et al., 2001; Roy et al., 2014).

## HnRNP A1 Function and Post-Translational Modifications

PTMs play a significant role in the functional diversity of hnRNP A1. HnRNP A1 undergoes phosphorylation, sumoylation, ubiquitination, PARylation, acetylation, methylation, and *O*-linked and *N*-linked β-N-acetylglucosaminylation (*O*-GlcNAcylation/*N*-GlcNAcylation), and all affect the location and functionality of hnRNP A1 (Figure 1B and Table 1). Since cellular location partially defines the function and activity of hnRNP A1, these effects are inextricably intertwined. Importantly, many of these modifications have been identified and characterized in the context of cancer biology and/or general stress responses, rather than in neurons or neurodegenerative disease, leaving us with important, unanswered questions and several avenues for future exploration.

**TABLE 1:** Post-translational modifications of hnRNP A1.

	Amino acid location	Mediator	Consequence of PTM
Phosphorylation	Serine 4	S6K2	Decreased IRES RNA binding, increased IRES translation (Roy et al., 2014)
	Serine 6	VRK1	Increased telomerase activation (Choi et al., 2012)
		S6K2	Decreased IRES RNA binding, increased IRES translation (Roy et al., 2014)
	Threonine 51	PERK	Destabilization of hnRNP A1 protein (Koo et al., 2016)
	Serine 95	DNA-PKcs	Reduced RNA binding (Sui et al., 2015)
	Serine 192	DNA-PKcs MNK1	Regulation of splicing activity (van der Houven van Oordt et al., 2000; Buxade et al., 2005; Guil et al., 2006; Ziaei et al., 2012; Sui et al., 2015)
	Serine 199	AKT	Decreased IRES RNA binding, translocation to cytoplasm (Martin et al., 2011)
	Serines 310-312	MNK1, MNK2	Reduced TNPO1 interaction resulting in cytoplasmic accumulation (Allemand et al., 2005) Increased cytoplasmic retention with canonical stressors, but not with T-cell activation (van der Houven van Oordt et al., 2000; Buxade et al., 2005; Guil et al., 2006; Ziaei et al., 2012) Facilitates interaction with stress granules under canonical stressors (Guil et al., 2006)
Methylation	Arginine 31	PRMT3	Asymmetrical di-methylation Reduced RNA binding, permitting increased translation of the mRNA (Hsu et al., 2018)
	Arginine 218, Arginine 225	PRMT5	Symmetrical di-methylation Increased IRES-mediated translation from HIV and HTLV-1 viruses (Gao et al., 2017; Barrera et al., 2020) Decreased RNA binding activity
	Arginine 206, Arginine 218, Arginine 225, Arginine 232	PRMT1	Asymmetrical di-methylation Reduction in ITAF activity (Wall and Lewis, 2017)
Ubiquitination	Lysine 3, Lysine 8, Lysine 15	TRAF6	K63-linked ubiquitination Induction of alternative splicing (Fang et al., 2017)
	Lysine 183, **Lysine 298^a^**	SPSB1 with Elongins B & C and Cullins 2 & 5	Unconventional K29-linked ubiquitination Induction of alternative splicing (Wang et al., 2017) Some cytoplasmic accumulation Reduced RNA binding
	*unknown*	USP7, USP5	De-ubiquitination of hnRNP A1, results in hnRNP A1 protein stabilization (Vashistha et al., 2020; Zhang et al., 2020)
Acetylation	Lysine 3, Lysine 52, Lysine 87	*unknown*	De-acetylation by SIRT1 results in reduced cellular proliferation (Yang et al., 2019)
Sumo-ylation	Lysine 183	Ubc9**^a^**	Re-shuttling to the nucleus from the cytoplasm after mRNA transport. Requires prior phosphorylation on Ser4/6 and the binding of 14-3-3 protein (Roy et al., 2014)
β-N-acetyl-glucosamin-ylation	Serine 22	OGT**^a^**	Increased interaction with TNPO1 and enhanced nuclear localization (Hart et al., 2011; Roth and Khalaila, 2017)
PAR-ylation	**Lysine 298^a^**	PARP1	Promotes hnRNP A1 cytoplasmic localization to stress granules (Duan et al., 2019) Increases association with TDP-43 (Duan et al., 2019)

*Amino acid numbering is based upon hnRNP A1 isoform A. ^a^Putative. ^b^Bolded residues are in the hnRNP A1 M9 nuclear localization sequence. S6K2, ribosomal protein S6 kinase 2; VRK1, vaccinia-Related Kinase 1; PERK, eukaryotic translation initiation factor 2 alpha kinase 3; DNA-PKcs, DNA-dependent protein kinase catalytic subunit; MNK1/2 MAP, kinase signal-integrating kinase 1; AKT, protein kinase B; PRMT1/3/5, protein arginine methyltransferase; TRAF6, TNF receptor-associated factor 6; SPSB1, SplA/Ryanodine receptor domain and SOCS box containing 1; USP5/7, ubiquitin specific peptidase; Ubc9 SUMO, conjugating enzyme; OGT, O-linked N-Acetylglucosamine (GlcNAc) transferase; PARP-1, poly(ADP-Ribose) polymerase 1.*

HnRNP A1 is considered a key component of the response to cellular stresses, and neuronal dysfunction and loss in neurodegenerative disease is mediated in part by cellular stress. The activities of hnRNP A1 in regulating RNA metabolism are likely to be its major function in responding to cell stress – altered RNA binding and modulation of alternative splicing, altered transportation to the cytoplasm with mRNAs, and a shift to facilitating cellular translation to cap-independent translation from internal ribosomal entry sites (IRESs). The majority of characterized PTMs are shown to regulate these hnRNP A1 activities and are broadly characterized in the context of canonical cellular stressors: heat shock, low-dose ultraviolet irradiation, osmotic and oxidative stress, and external signaling factor stimulation. From these, we can extrapolate outcomes in response to other inputs and stressors, including protein aggregate accumulation or chronic inflammation that are associated with neurodegenerative disease.

### HnRNP A1 and Transcription

**FIGURE 2:**
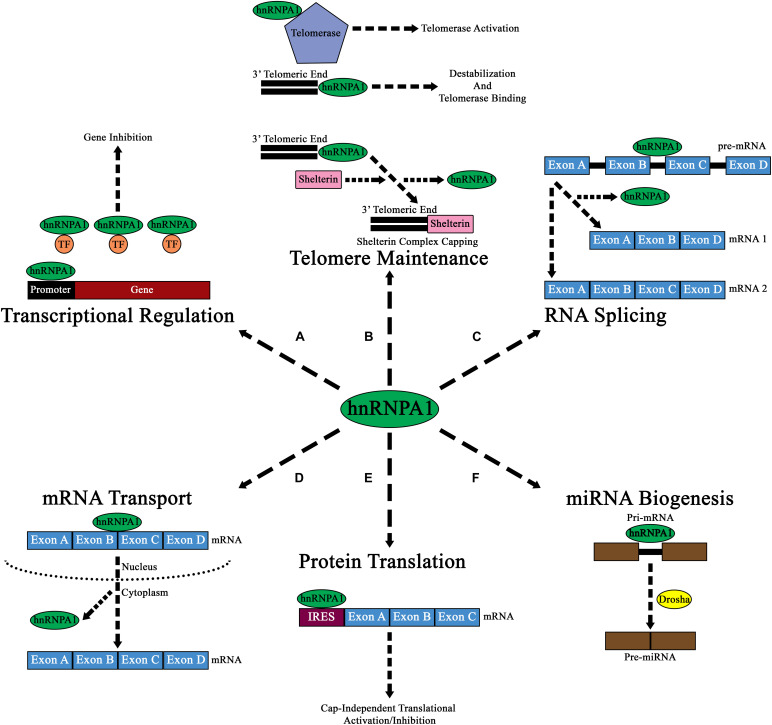
Diagram of the major cellular functions of hnRNP A1. **(A)** Nuclear hnRNP A1 can regulate transcriptional expression by either promoter regulation, or by binding and sequestering transcription factors (TF; orange circles). **(B)** hnRNP A1 can also regulate telomeric elongation and capping either via telomerase binding and activation, direct 3’ telomeric end binding, or by aiding in the binding of Shelterin complex proteins. The results of these interactions are to prevent NHEJ and telomere degradation. **(C)** Pre-mRNA splicing is a major component of hnRNP A1 nuclear function, and its binding can result in the formation of various mRNA isoforms from a single pre-mRNA transcript. **(D)** mRNA transport from the nucleus to the cytoplasm has been shown to be a major function of hnRNP A1, where hnRNP A1 directs mRNA to ribosomes for cap-dependen translation. **(E)** In addition to directing mRNA to ribosomes for cap-dependent mRNA translation in the cytoplasm, hnRNP A1 can also bind to IRES elements within mRNA and influence cap-independent translational activation or inhibition. **(F)** hnRNP A1 has been shown to influence the nuclear formation of pre-miRNAs from pri-miRNAs. While the exact mechanism of how hnRNPA1 affects this pathway is unknown, it is theorized that hnRNP A1 may influence Drosha (yellow circle) binding to pri-miRNA.

Initial reports described hnRNP A1 as a DNA binding protein that influences RNA transcription through its binding to promoters, whereby its interaction either suppressed or activated transcription, depending on the gene of interest (Figure 2A; Lau et al., 2000; Campillos et al., 2003; Chen et al., 2003; Das et al., 2004; Xia, 2005). The mechanisms how hnRNP A1 affects transcriptional regulation are predominantly divided between two lines of evidence. Firstly, it has been shown that hnRNP A1 regulates promoter suppression by mediating the activity of transcriptional factors through protein-protein interactions with its PrLD domain (Hay et al., 2001). Other studies have shown that hnRNP A1 regulates promoter activation by binding to and destabilizing G-quadruplex structures within the promoters of genes (Takimoto et al., 1993; Fukuda et al., 2002; Paramasivam et al., 2009). This latter effect depends upon recognition of the RGG domain and subsequent binding to G-quadruplex DNA by interacting with loop nucleotides of the G-quadruplex structure (Paramasivam et al., 2009; Ghosh and Singh, 2018; Liu and Xu, 2018). This interaction then allows the RRMs of hnRNP A1 to destabilize the G-quadruplex structure and stabilize the unfolded form of the DNA through its interaction with single-stranded DNA (Paramasivam et al., 2009; Ghosh and Singh, 2018; Liu and Xu, 2018).

Dysregulated cellular senescence is also implicated in the progression of several neurodegenerative disorders [reviewed in Kritsilis et al. (2018) and Martinez-Cue and Rueda (2020)], and global hnRNP A1 knockdown results in cellular senescence (Shimada et al., 2009), as indicated in its role in cell cycle progression and senescence. Transcription of oncogenes, like Kirsten Rat Sarcoma Viral Proto-Oncogene (*KRAS)* and Transformer 2 Beta Homolog (*TRA2B)*, are promoted by hnRNP A1 binding to a G-quadruplex structure in the genes’ promoters, utilizing hnRNP A1 DNA binding activity (Paramasivam et al., 2009; Nishikawa et al., 2019). Promotion of *KRAS* transcription has recently been shown to be regulated by the cell stress response regulator poly (ADP-ribose) polymerase (PARP-1), which itself PARylates hnRNP A1 (discussed below), although the impact of hnRNP A1 PARylation on *KRAS* transcription, and the function of KRAS expression in the context of oxidative stress are currently unclear (Table 1) (Duan et al., 2019; Cinque et al., 2020). Additionally, hnRNP A1 stabilizes *SIRT1* mRNA (Wang et al., 2016); increased abundance of its protein product Sirtuin/SIRT1 prevents progression to cellular senescence, but also de-acetylates hnRNP A1 protein, which negatively regulates oncogenic cell proliferation by modulating alternative splicing of pro-survival mRNAs like *PKM* (Table 1) (Yang et al., 2019). Since both de-acetylation and acetylation at different sites on hnRNP A1 result in similar consequences (see Table 1), the regulation of cellular senescence and proliferation by hnRNP A1 by these processes may require additional experimental exploration.

### HnRNP A1 and Telomeres

Further insights into hnRNP A1/DNA interactions have shown that hnRNP A1 also affects telomeric metabolism, in a multifaceted mechanism (Figure 2B). Firstly, hnRNP A1 helps destabilize the G-rich extension at the 3′ telomeric end through interactions with its RRMs to promote the association of telomerase (Ghosh and Singh, 2018). Additionally, hnRNP A1 binds to the single-stranded DNA and RNA component of telomerase and promotes its activation at the telomere (Wang et al., 2019). HnRNP A1 also promotes the Shelterin protein complex capping of telomeres through its binding with DNA-dependent protein kinase catalytic subunit (DNA-PKcs) (Table 1). HnRNP A1 is a direct substrate for DNA-PKcs, and phosphorylated hnRNP A1 promotes Shelterin complex formation by initially promoting the association of protection of telomeres 1 (POT1) protein to telomeres, that then associates with other Shelterin proteins to form a complex (Figure 2B and Table 1; Sui et al., 2015).

Phosphorylation is also key to how hnRNP A1 modulates telomerase activity during cell cycle progression. HnRNP A1 binds both G-quadruplex telomere DNA and telomere repeat-containing RNA (TERRA), as well as telomerase itself, and further modifies the balance between telomerase regulatory molecules RPA (replication protein A) and POT1 (Table 1; Fiset and Chabot, 2001; Flynn et al., 2011; Choi et al., 2012; Redon et al., 2013; Ghosh and Singh, 2018). HnRNP A1 has been found to have both negative and positive effects on telomerase activity and telomere length (Redon et al., 2013); some of this has been shown to be due to its phosphorylation. In G2/M phase cells, hnRNP A1 is phosphorylated at the SQ motif of Ser95 and Ser192 by DNA-PKcs, and at Ser6 by vaccinia-related kinase 1 (VRK1) (Table 1; Choi et al., 2012; Sui et al., 2015); VRK1-mediated phosphorylation appears to result in hnRNP A1 promoting telomerase activity, while phosphorylation by DNA-PKcs instead promotes hnRNP A1-mediated protection of telomeres from stimulating DNA damage responses (Table 1). In this instance, phosphorylated hnRNP A1 functions in a pro-survival mechanism that prevents the unique structure of DNA telomeres exposed during the G2/M phase of cell cycle progression from aberrantly triggering DNA damage responses and cell death. Overall, the regulation of hnRNP A1 by several kinases affect cell survival through cell cycle progression. Its direct impact on neurodegenerative disease, however, remains to be explored.

### HnRNP A1 and RNA Splicing

In addition to affecting RNA transcription and telomeric metabolism, hnRNP A1 plays a significant role in RNA splicing (Figure 2C). HnRNP A1 is an essential component of the spliceosome, where it contributes to both constitutive and alternative mRNA splicing. HnRNP A1 acts as a negative *cis* splicing element, binding to either exonic splicing silencer (ESS) elements, or to intronic splicing silencer (ISS) elements found within pre-mRNA, leading to blocked exon recognition or promotion of exon exclusion in mRNA (Han et al., 2005). In neurodegenerative diseases, it has been theorized that altered hnRNP A1 binding to ESS or ISS elements within pre-mRNA may lead to pathogenesis. As described later in this review, altered splicing activity of hnRNP A1 to targets such as *SMN2*, *APP*, *TAU*, *MAG*, and even its own pre-mRNA, may lead to neurodegeneration.

The splicing process of hnRNP A1 is tightly regulated by a variety of PTMs that generally disrupt hnRNP A1 RNA binding activity – a common theme for controlling hnRNP A1 function – resulting in alternative splicing of hnRNP A1-regulated mRNAs. This has been characterized in the context of viral mRNAs as well as host mRNAs, and through a variety of stimuli. An early characterization utilized the Adenovirus E1A mRNA as a splicing reporter, identifying that activation of p38-MAPK through osmotic and UV stressors resulted in hnRNP A1 phosphorylation and alternative splicing of the viral mRNA (Table 1; van der Houven van Oordt et al., 2000). This also results in accumulation of hnRNP A1 in the cytoplasm, another common theme of the effects of PTMs of hnRNP A1 (van der Houven van Oordt et al., 2000). Later works indicate that it is likely MAP kinase signal-integrating kinase 1 (MNK1), a downstream kinase, that carries out p38-MAPK-stimualted phosphorylation (Table 1; Buxade et al., 2005; Guil et al., 2006; Ziaei et al., 2012).

HnRNP A1 alternative splicing activity has also been shown to be regulated by ribosomal protein S6 kinase 2 (S6K2) phosphorylation of hnRNP A1 Ser6 in cancer (discussed below) (Table 1; Sun et al., 2017). Non-canonical ubiquitination of hnRNP A1, however, has also been characterized as a regulator for hnRNP A1-mediated alternative splicing. Wang et al. determined that the adaptor protein SplA/Ryanodine receptor domain and SOCS box containing 1 (SPSB1) coordinates K29-linked ubiquitination of hnRNP A1 in response to EGF stimulation at Lys183 and Lys298 requires the Elongin B/C-Cullin 2/5 E3 ubiquitin ligase complexes (Table 1; Wang et al., 2017). This unusual ubiquitination reduces hnRNP A1 affinity for RNA and permits alternative splicing of rac family small GTPase 1 *(RAC1)* mRNA to the *RAC1B* isoform, as well as family with sequence similarity 13 member B (*FAM13B)*, muscleblind like splicing regulator 1 *(MBNL1)*, and RNA binding motif protein 10 *(RBM10)* (Wang et al., 2017). The *b* splice isoform of the Rac1 protein is associated with increased motility in cancer cells, while FAM13B function remains poorly characterized, and Rbm10 and MBNL1 are themselves both important regulators of mRNA splicing (Wang et al., 2017; Bergsma et al., 2018). In parallel, Fang et al. characterized hnRNP A1 in response to Toll-like receptor (TLR) signaling due sensing pathogen-associated molecules like bacterial lipopolysaccharides (Fang et al., 2017). This inflammatory signal stimulates TNF Receptor-Associated Factor 6 (TRAF6), an E3 ligase that is also a transducer of TLR signaling to ubiquitinate the RRM1 region of hnRNP A1 with K63-linkages, again resulting in reduced RNA binding to facilitate alternative splicing of many mRNAs (Fang et al., 2017). Overall, PTMs of hnRNP A1 that impact its splicing activities cause hnRNP A1 to release its protective binding at non-canonical exons, allowing alternative splicing using those exons and producing alternate forms of select mRNAs.

### HnRNP A1, RNA Trafficking and Translation

HnRNP A1, however, can also affect mRNA translation, as it is an internal ribosome entry site (IRES)-trans activating factor (ITAF) that binds IRES sequences, and regulates ribosomal entry and/or transcript reading (Bonnal et al., 2005; Jo et al., 2008; Martin et al., 2011). Specifically, studies have shown that hnRNP A1 can regulate the IRES dependent translation of mRNAs that encode proteins related to apoptosis and proliferation (Bonnal et al., 2005; Lewis et al., 2007; Jo et al., 2008; Martin et al., 2011; Damiano et al., 2013).

HnRNP A1 has been identified to act as an IRES *trans*-activating factor (ITAF) to many cellular IRES containing mRNAs, including mRNAs for oncogenes and cell cycle regulators like Myc Proto-Oncogene Protein (c-Myc), Cyclin D, B-cell lymphoma-extra-large (BCL-XL), fibroblast growth factor 2 (FGF-2), and X-linked inhibitor of apoptosis (XIAP) (Bonnal et al., 2005; Martin et al., 2011; Roy et al., 2014). Cellular switching to IRES-mediated (cap-independent) translation can be initiated in response to cellular stressors, during cell cycle progression or growth signaling, or under aberrant signaling conditions like oncogenesis, and functions to permit production of select proteins while global cap-dependent translation is halted (Godet et al., 2019; Yang and Wang, 2019). HnRNP A1 binding to IRES sequences in mRNAs is thought to facilitate trafficking of the mRNAs to the cytoplasm, where subsequent phosphorylation of hnRNP A1 results in the protein releasing the IRES, allowing cap-independent translation to initiate (Martin et al., 2011; Roy et al., 2014; Sun et al., 2017). As an example, FGF2 stimulates the kinase S6K2 to phosphorylate Ser6 of RNA-bound hnRNP A1 in the nucleus, driving association with nuclear RNA export factor 1 (NXF1) and transport from the nucleus with IRES-containing mRNAs like *BCL-XL* and *XIAP* (Table 1; Roy et al., 2014). After cytoplasmic release of the mRNAs, the 14-3-3 protein binds phosphorylated hnRNP A1 and acts as an adaptor for an unknown Small Ubiquitin Like Modifier (SUMO) E3 ligase; SUMOylated hnRNP A1 then efficiently returns to the nucleus (Roy et al., 2014). Several other kinases have been implicated in similar regulation [e.g., protein kinase B (PKB; AKT), MNKs; see Table 1], and it is likely that different kinases carry out this phosphorylation dependent upon the stressor or stimulus (Martin et al., 2011; Roy et al., 2014; Sun et al., 2017). Particularly, S6K2 expression is associated with response to growth factor signaling and tumorigenesis, and its phosphorylation of hnRNP A1 at Ser4 and Ser6 may be aberrant in differentiated cells (Roy et al., 2014; Sun et al., 2017).

Protein arginine (Arg) methyltransferase PRMT5 also regulates hnRNP A1 promotion of IRES-dependent translation of both HIV and HTLV-1 viral RNAs, and several cellular mRNAs through symmetrical di-methylation at Arg218 and Arg225 of hnRNP A1 (Table 1; Gao et al., 2017; Barrera et al., 2020). Methylation at these sites appears to have a similar impact as phosphorylation, resulting in decreased RNA binding and freeing IRES RNA structures to permit assembly of translation initiation factors (Gao et al., 2017; Barrera et al., 2020); methylation at Arg31 by PRMT3 has a similar effect, reducing hnRNP A1 binding to the cellular ATP binding cassette subfamily G member 2 (*ABCG2)* mRNA and increasing its protein abundance (Hsu et al., 2018). It is not clear from these studies whether these methylation events occur in the nucleus or cytoplasm, as the subcellular localization of PRMTs are highly variable (Tang et al., 1998; Frankel and Clarke, 2000; Rho et al., 2001; Lee et al., 2005; Meyer et al., 2007; Herrmann et al., 2009). Nonetheless, it is likely that PRMT5 methylation occurs in the cytoplasm, as decreased RNA binding in the nucleus would affect mRNA transport to the cytoplasm, where the assembly of translational machinery occurs. Alternatively, asymmetrical methylation of hnRNP A1 by PRTM1 at Arg206, 218, 225, and 232 has been shown to reduce IRES-mediated translation (Table 1; Wall and Lewis, 2017). When Hartel et al. screened the human “methylome” by a combination of biochemical methods with and without PRTM1 knockdown, they identified a switch in methylation of hnRNP A1 Arg206, from asymmetrical to symmetrical methylation, and suggest that this may be evidence of mutually antagonistic activities of PRTM1 and PRTM5 in regulating hnRNP A1 ITAF activity (Table 1; Hartel et al., 2019).

HnRNP A1 also modulates translation through binding in the 3′ UTR of mRNAs – this function was originally defined by hnRNP A1 binding in AU-rich elements (AREs) in mRNAs and reducing translation activity of the mRNAs. The mRNA for the inflammatory cytokine TNFα contains an ARE, as do many other cytokines’ mRNAs; particularly, *TNF* has been shown to be bound, and its translation suppressed, by hnRNP A1. Buxadé et al. carefully defined the mechanism by which suppression is lifted and found that mRNA circularization brings 3′-bound translational regulatory factors into proximity to the translation initiation complex, permitting activated MNK1 to mono-phosphorylate hnRNP A1 at one of Ser310, Ser311, or Ser312 (Table 1; Buxade et al., 2005). Phosphorylation of hnRNP A1 in this poly-serine tract (comprised of Ser308-313, and Ser316) causes it to release the *TNF* mRNA, and de-repress its translation (Table 1; Buxade et al., 2005). Canonical stress-induced MNK1-mediated phosphorylation of hnRNP A1 also requires hnRNP A1 RNA-binding activity (Table 1). This suggests that hnRNP A1 is itself a better MNK1 substrate when bound to RNA; RNA-bound hnRNP A1 is also a better substrate for DNA-PKcs-mediated phosphorylation, although this latter activity may be predominantly associated with hnRNP A1 splicing activity (Zhang et al., 2004). While the key players may change depending upon stimulus, the core mechanism is likely common across hnRNP A1-regulated mRNA translational suppression.

### HnRNP A1 Cellular Localization

HnRNP A1 also functions to transport mRNAs from the nucleus to the cytoplasm (Figure 2D) and affects mRNA translation (Figure 2E). The majority of characterized PTMs that regulate hnRNP A1 localization do so directly in the context of the protein’s DNA or RNA-regulating functions. Particularly, several of the modifications that affect hnRNP A1 translation-modulating activities are driven by cellular stress responses and promote translocation to the cytoplasm. PARylation of hnRNP A1 by PARP-1 has been recently identified to occur in neurons in response to stress; PARP-1 activation broadly promotes SG formation, and PARylation of hnRNP A1 results in its nuclear depletion and increased accumulation in SGs (Table 1; Duan et al., 2019). Interestingly, Duan et al. note that hnRNP A1 has a putative PAR binding site between RRM1 and RRM2; while they find that hnRNP A1 PARylation and this site are both required for interaction with TDP-43 (another protein widely involved in neurodegenerative disease) in SGs, they do not define whether this may affect hnRNP A1 self-interaction (Table 1; Duan et al., 2019). In addition, unlike most of the research described above, Duan et al. monitor resolution of the stress response, finding that increased PARylation of hnRNP A1 results in delayed SG disassembly (Duan et al., 2019). This outcome provides a link between cellular stressors, PTMs of hnRNP A1, and a dysregulated stress response that could lead to long-term pathology.

In contrast, *O*-GlcNAcylation appears to modulate hnRNP A1 localization in the brief window where it is not interacting with or regulating RNA metabolism and appears to play a role in restoring hnRNP A1 nuclear localization. Roth and Khalaila have identified a single *O*-GlcNAc modification at Ser22 and demonstrate that *O*-GlcNAcylation increases hnRNP A1 association with TNPO1, the nuclear transport protein that returns hnRNP A1 to the nucleus to resume regulation of RNA production (Table 1; Roth and Khalaila, 2017). They do not define a cellular stimulus that promotes this modification but note that phosphorylation and *O*-GlcNAcylation can function antagonistically, such that *O*-GlcNAcylation may be potentiated by some of the hnRNP A1 phosphorylation states described above as a means of restoring hnRNP A1 nuclear localization and cellular homeostasis (Hart et al., 2011; Roth and Khalaila, 2017). Interestingly, SUMOylation in response to 14-3-3 binding of phosphorylated hnRNP A1 described above seems to serve the same or a similar purpose, marking RNA-depleted hnRNP A1 for return to the nucleus (Table 1; Roy et al., 2014).

Separately, increased MNK1 activity has been shown to promote hnRNP A1 localization to the cytoplasm during cellular senescence, although it is not yet clear whether MNK1 directly phosphorylates hnRNP A1 to facilitate this (Table 1; Ziaei et al., 2012). However, MNK1 has been shown to phosphorylate hnRNP A1 in NIH3T3s, and specifically at Ser192 and Ser310-311-312 in activated T-cells (Table 1; van der Houven van Oordt et al., 2000; Buxade et al., 2005). Additionally, activity of p38 MAP kinase (an upstream activator of MNK1) has resulted in hnRNP A1 phosphorylation and localization to the cytoplasm during cell stress (discussed above) and senescence (Table 1). Again, it is not clear whether p38 MAPK directly phosphorylates hnRNP A1, but overall activation of the MAPK signaling cascade seems to result in hnRNP A1 phosphorylation, translocation to the cytoplasm, and promotion of cellular senescence (Shimada et al., 2009).

A large proportion of the modifications described above should result in temporary alterations to hnRNP A1 function, including mRNA splicing, transport, and translation. These data provide the first means to resolve these responses and restore homeostatic function, and it is likely that dysregulation of these resolution processes can themselves be pathological.

### HnRNP A1 Stability and Other Functions

Turnover of hnRNP A1 protein is mediated by ubiquitination, like much of the mammalian proteome. Wen et al. have recently identified a long non-coding RNA, *ANCR*, that prevents ubiquitination of hnRNP A1, resulting in its stabilization, but whether *ANCR* prevents ubiquitination or promotes de-ubiquitination of hnRNP A1, and by what mechanisms, is unknown (Wen et al., 2020). Zhang et al. and Vashistha et al. have separately characterized two de-ubiquitinating enzymes [Ubiquitin Specific Peptidase 5 and 7 (USP5 and USP7), respectively] that remove classical K48-linked ubiquitin degradation markers and prevent hnRNP A1 degradation; both of these studies were done in the context of oncogenesis and chemotherapy resistance, and indicate a role of stabilized hnRNP A1 in cellular survival (Table 1; Vashistha et al., 2020; Zhang et al., 2020). In contrast, Koo et al. have found that eukaryotic translation initiation factor 2 alpha kinase 3 (PERK)-mediated phosphorylation of hnRNP A1 at Thr51 results in hnRNP A1 degradation in response to endoplasmic reticulum stress (e.g., exposure to tunicamycin or thapsigargin), although the mechanism of this destabilization is not understood (Table 1; Koo et al., 2016). HnRNP A1 regulates the processing of at least two known microRNAs (miRNAs), pri-miR-18a and pri-Let-7a, which themselves modulate mRNA stability and translation (Figure 2F). HnRNP A1 binds to primary miRNA (pri-mRNA) in the nucleus and is thought to promotes cleavage into pre-miRNA by promoting Drosha protein binding (Guil and Caceres, 2007; Michlewski and Caceres, 2010; Michlewski et al., 2010). Thus, when hnRNP A1 is depleted, the authors find a reduction in miR-18a processing and activity in hepatic stellate cells, broadly increasing the translation of several targets and promoting fibrotic signaling in the liver (Koo et al., 2016). Clearly, the consequence of global changes to hnRNP A1 abundance vary by cell type and stimulus; there is a large body of data from hnRNP A1 overexpression and knockdown experiments, but metrics of interest are rarely defined compared to hnRNP A1 abundance. However, a recent paper by Duan et al. (discussed above) uses PARylation inhibition to abrogate the effects of long-term hnRNP A1 overexpression in neuron-like cells, suggesting that long-term hnRNP A1 accumulation likely leads to cell stress signaling mediated by PARylation in a neuronal context (Duan et al., 2019).

## HnRNP A1 and Neurodegenerative Diseases

**FIGURE 3:**
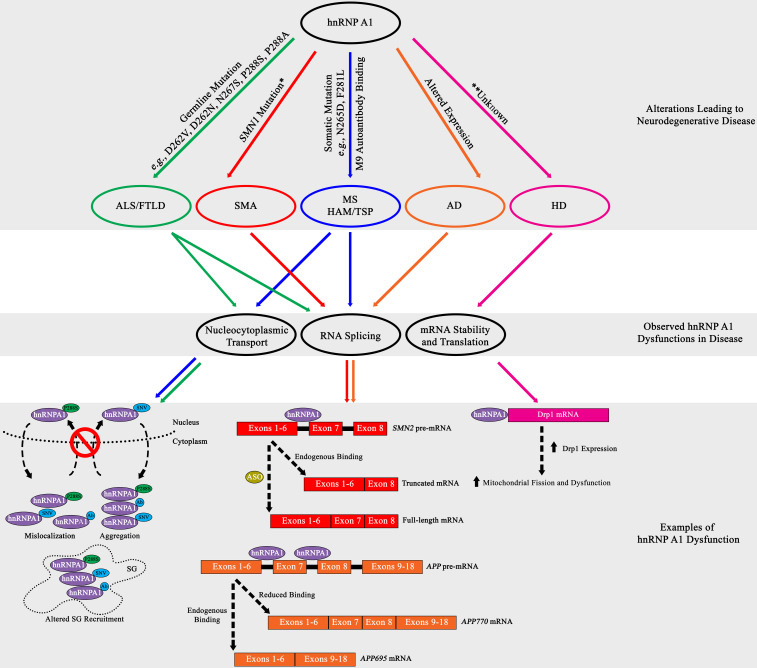
Summary diagram of hnRNP A1 dysfunctional pathways leading to neurodegenerative disease pathogenesis. Top Grey Box: HnRNP A1 alterations that are disease specific (ALS/FTLD, green; SMA, red; MS and HAM/TSP, blue; AD, orange; HD, pink) lead to pathogenesis. Middle Gray Box: Commonly observed hnRNP A1 dysfunctions in neurodegenerative diseases are nucleocytoplasmic transport deficits (leading to protein mislocalization, aggregation and altered SG recruitment), dysregulated RNA splicing, and modification of target mRNA stability. Bottom Gray Box: Selectively highlighted examples of hnRNP A1 dysfunction in neurodegenerative disease. Each dysfunctional mechanism is colour coded for the disease it refers to. Refer to the individual neurodegenerative disease sections for more information. Ab = antibody. *Dysregulation of the SMN1 gene precedes the effects of hnRNP A1 splicing.

A consequence of the toxic accumulation of proteins within the neuronal milieu of neurodegenerative disease is perturbation in RNA metabolism [reviewed in Liu et al. (2017)]. While the contribution of hnRNP A1 to the disturbance of RNA metabolism and its subsequent consequences has been characterized in cancer and viral immunology, the role of hnRNP A1 in altered RNA metabolism in neurodegenerative disease is less well understood. This lack of understanding is in part due to the complicated systemic nature of neurodegenerative diseases, where physical brain architecture, interactions with the immune system, and multiple glial cell types all contribute to disease progression, making modeling difficult. Identification of molecular players has historically begun with detection of associations between heritable or spontaneous mutations and disease, only then leading to careful characterization in animal models and cell culture. Studies in amyotrophic lateral sclerosis (ALS), frontotemporal lobar degeneration (FTLD), multiple sclerosis (MS), spinal muscular atrophy (SMA), Alzheimer’s disease (AD) and Huntington’s disease (HD) all show compelling evidence that suggests hnRNP A1 may influence neurodegenerative disease pathogenesis (Table 2 and Figure 3). However, our understanding of the molecular mechanisms – including regulation by PTMs – by which hnRNP A1 contributes to these diseases remains incomplete.

**TABLE 2:** Proposed mutations and effects of hnRNP A1 dysfunction in neurodegenerative diseases.

Neurodegenerative Disease	Disease-associated mutations	Proposed hnRNP A1 altered function	Disease alteration
ALS/FTLD	**^a^** p.D262V p.D262N p.N267S p.P288S p.P288A	Nucleocytoplasmic transport	Prolonged LLPS leading to increased protein-protein interactions.
		Nucleocytoplasmic transport	Increased cytoplasmic insoluble hnRNP A1 aggregation.
		Nucleocytoplasmic transport	Altered SG dynamics, leading to stable and prolonged SG formation.
		RNA splicing	Altered interaction with TDP-43, leading to increased longer *hnRNP A1* splice variant formation.

SMA		RNA splicing	Formation of unstable and truncated SMN1 protein from *SMN2* RNA.

MS and HAM/TSP	**^b^** p.S252N	Nucleocytoplasmic transport	Antibodies produced by an immune response bind to hnRNPA1, sequestering it in the cytoplasm and leading to increased insoluble hnRNP A1 aggregation.
	p.S259G	RNA splicing	Cytoplasmic mislocalization of hnRNPA1 leading to the dysregulation of spastin, spartin and paraplegin splicing.
	p.N265D		
	p.F263L p.F273L	Nucleocytoplasmic transport	Somatic mutations in hnRNP A1 lead to increased cytoplasmic retention and increased insoluble hnRNPA1 aggregation.
	p.P275S p.M276L p.K277N p.N280S p.F281L p.R284G p.S285G p.Y295C p.F296L p.P299L p.R300S p.N301D p.N301S p.S308P p.S313G p.Y314C p.Y314H p.G317D p.G317S p.R318G p.R319G p.F320L	RNA splicing	Altered *S-MAG/L-MAG* mRNA ratio, leading to an increase in *S-MAG* mRNA and protein expression. No direct evidence in MS, but data from EAE mice show a depletion in MAG protein in the spinal cord.

Alzheimer’s disease		RNA splicing	Increased generation of longer *APP* splice variant, leading to increased amyloid-β formation.
		RNA splicing	Increased generation of *mRAGE* mRNA, leading to an increase in mRAGE expression.
		RNA splicing	Altered *4R-Tau/3R-Tau* mRNA ratio, leading to tauopathy.

Huntington’s disease		mRNA stability	Increased expression of Drp1, leading to increased mitochondrial fragmentation and cell death.

*^a^Germline Mutations. ^b^Somatic Mutations.*

### Amyotrophic Lateral Sclerosis (ALS) and Frontotemporal Lobar Degeneration (FTLD)

Insights into ALS and FTLD have shown that genetic mutations and altered expression of hnRNP A1 may influence pathogenesis. Mutations in hnRNP A1 have been associated with forms of ALS and FTLD and have been suggested to affect hnRNP A1 insoluble protein aggregation. HnRNP A1 is hypothesized to promote insoluble protein aggregation through mutations found in its PrLD, resulting in alterations in protein-protein interactions, including self-association and fibrillization propensity. In a study on a family with multisystem proteinopathy (MSP), a rare syndrome that includes inclusion body myopathy with FTLD, Paget’s disease of bone and ALS, the authors reported three distinct mutations in hnRNP A1, p.D262V, p.D262N and p.N267S (Kim et al., 2013). These mutations were found in the PrLD of hnRNP A1 and were shown to both increase the recruitment of hnRNP A1 to SGs and accelerate hnRNP A1 fibrillization and the formation of prionogenic protein accumulations, by both deregulating and accelerating the nucleation and polymerization process of hnRNP A1 (Figure 3; Kim et al., 2013).

The downstream effects of altered hnRNP A1 protein dynamics are still currently under investigation, but a prevailing theory is that SG biology is a likely target of dysregulation. This theory is supported by data that demonstrates that a genetic missense mutation in the PrLD of hnRNP A1 found in ALS patients (c.862.1018C > T/p.P288S) resulted in the cytoplasmic mislocalization of hnRNP A1 and the formation of cytoplasmic inclusions (Figure 3; Liu et al., 2016). The authors further showed that hnRNP A1 inclusions colocalized with SGs but did not elaborate further on the effect of hnRNP A1 inclusions on SG dynamics (Figure 3; Liu et al., 2016). Interestingly, these results were similarly replicated in a separate study where the authors discovered the familial ALS genetic missense hnRNP A1 mutation p.P288A and described its propensity to also form cytoplasmic inclusions that colocalized with SGs (Figure 3; Naruse et al., 2018). Further, a current study under review has demonstrated that the p.P288A and p.D262V mutations, as well as newly found mutations p.G304Nfs^∗^3, p.^∗^321Eext^∗^6 and p.^∗^321Qext^∗^6, have altered self-aggregation properties, undergo reduced LLPS, and in the case of p.P288A and p.D262V, attenuate both SG formation and dissociation properties (Beijer et al., 2021). A subset of these hnRNP A1 mutations introduce new potential phosphorylation sites that may aberrantly regulate hnRNP A1 functionality, contributing to disease (Blom et al., 1999, 2004).

As mentioned above, TDP-43 interacts with hnRNP A1 through its PrLD and influences the splicing activity of hnRNPA1, as well as modulates *HNRNP A1* mRNA splice variant formation (Deshaies et al., 2018). Specifically, research has shown that depleting endogenous TDP-43 with siRNA in HeLa cells increases the retention of exon 7B in *HNRNP A1* mRNA, resulting in increased *HNRNP A1-B* isoform expression (Deshaies et al., 2018). The result of this increase was the generation of toxic cytoplasmic hnRNP A1 aggregates that were also comparable in size to those found in cells expressing the ALS hnRNP A1 mutation D262V (Deshaies et al., 2018). While this study focuses primarily on the effect of TDP-43 perturbation on altered hnRNP A1 dynamics, mutational alterations of hnRNP A1 might similarly influence TDP-43 interaction. Furthermore, since TDP-43 is suggested to affect both ALS and FTLD disease progression, it is possible that hnRNP A1 may play a similar role.

### Spinal Muscular Atrophy (SMA)

The stringent regulation of survival of motor neuron 1 (*SMN1*) RNA splicing is essential to the survival of α-motor neurons in the anterior horn of the spinal cord and has been shown to be significantly influenced by hnRNP A1. Briefly, the homozygous loss, interruption, or autosomal recessive mutation in the *SMN1* gene culminates in the reduced expression of SMN1 protein, resulting in SMA progression (Lefebvre et al., 1995, 1997). While *SMN1* deficiency is partially compensated for by the *SMN2* gene, a near identical copy of the *SMN1* gene, *SMN2* cannot fully compensate for *SMN1* genetic loss (Lefebvre et al., 1995, 1997). Research has established that although *SMN2* is transcribed comparably to *SMN1*, a silent genetic mutation in *SMN2* (c.840C > T) transforms a 3′ splice site ESE element within exon 7 into an ESS element, resulting in the translation of an unstable, truncated SMN1 protein due to the majority of *SMN2* mRNA lacking exon 7 (Cartegni and Krainer, 2002; Kashima and Manley, 2003). Research has established that hnRNP A1 binds to the ESS element found in *SMN2*, influencing the exclusion of exon 7, leading to SMN1 deficiency and disease pathogenesis (Figure 3; Kashima et al., 2007; Doktor et al., 2011). Binding of hnRNP A1 has been theorized to either directly block the 3′ splice site, or after initial binding, propagate along the exon or along the RNA polypurine tract to inhibit spliceosomal recognition and recruitment (Figure 3; Doktor et al., 2011).

Additional research suggests that hnRNP A1 binds to an ISS element in *SMN2* RNA resulting in its alternative splicing (Figure 3; Beusch et al., 2017). While the regulatory effects of phosphorylation on hnRNP A1-mediated alternative splicing have been characterized for other mRNAs, *SMN2* has yet to be explored in this context. The authors of this study indicate that both RRMs of hnRNP A1 bind to an intron 7 bipartite ISS element in *SMN2* RNA, in a cumulative and directional manner, with RRM2 contacting a 5′ motif and RRM1 contacting a 3′ motif, resulting in exon 7 exclusion (Beusch et al., 2017). In parallel, another study treated SMA fibroblast cells with the histone deacetylase (HDAC) inhibitor valproic acid and showed a decrease in hnRNP A1 protein expression that coincided with an increase in exon 7 *SMN2* transcription (Harahap et al., 2012).

The studies presented above suggest that targeting hnRNP A1 alternative splicing activity may have therapeutic value. In this regard, to date the most successful research on improving the translation of stable, full-length SMN1 through hnRNP A1 mediation was observed in a series of studies that utilized anti-sense oligonucleotide (ASO) blocking of the bipartite ISS element, thus blocking hnRNP A1 binding, which showed an enhanced inclusion of *SMN2* exon 7 by approximately 90% (Hua et al., 2007, 2008). The collective information from these and associated studies has led to the development of Nusinersen (Spinraza^®^), an ASO treatment that was FDA approved in 2016 for the treatment of infantile- and later-onset SMA (Finkel et al., 2017; Mercuri et al., 2018).

### Autoimmune Diseases of the Central Nervous System (CNS): Multiple Sclerosis (MS) and Human T-Lymphotropic Virus Type 1 (HTLV-1) Associated Myelopathy/Tropical Spastic Paraparesis (HAM/TSP)

Studies of MS and HAM/TSP have implicated hnRNP A1 dysfunction as a contributor to disease pathogenesis. Previously described as a predominantly demyelinating disease, MS is now considered both a disease of inflammatory-mediated demyelination and neurodegeneration (loss or damage to neurons and axons) (Peterson and Fujinami, 2007). These pathologic features are also present in HAM/TSP, which resembles progressive forms of MS clinically, but unlike MS, has been shown to be caused by infection with HTLV-1 (Jernigan et al., 2003; Lee et al., 2008, 2012).

Our lab showed that molecular mimicry contributed to the pathogenesis of HAM/TSP via cross-reactive antibodies between HTLV-1 and neuronal antigens. Specifically, we initially reported that IgG isolated from HAM/TSP patients and a monoclonal antibody (Mab) to HTLV-1 tax (the regulatory region of HTLV-1) stained neurons in human brains from healthy controls (Levin et al., 1998). This suggested that there was molecular mimicry between the viral and host proteins recognized by the antibodies. HAM/TSP IgG immunoreacted with a 33-38 kDa protein isolated from human neurons and the immunoreactive host protein was identified as hnRNP A1 by matrix assisted laser desorption ionization (MALDI) mass spectroscopy (NM). Further investigation revealed that both the tax Mab and HAM/TSP IgG immunoreacted with hnRNP A1 (Levin et al., 2002a; Lee S.M. et al., 2006). The epitope of the monoclonal antibody mapped to tax 346-353 (KHFRETEV) and that of HAM/TSP IgG within hnRNP A1-M9 (293-GQYFAKPRNQGG-304) (Levin et al., 2002b; Lee S.M. et al., 2006). The tax Mab also immunoreacted with the identical hnRNP A1-M9 epitope (Lee S.M. et al., 2006). Pre-absorption of the tax Mab with hnRNP A1 inhibited tax Mab staining for human neurons and immunoreactivity with hnRNP A1 by Western blot. Taken together, these data indicate molecular mimicry between hnRNP A1 and HTLV-1-tax. There was little primary sequence identity between the viral and host epitopes. This is common, considering that molecular mimicry due to immunologic cross-reactivity as shown by these and other data have increased biological significance compared with mimics defined by primary sequences (Oldstone, 1998; Albert and Inman, 1999; Gran et al., 1999; Lee and Levin, 2008). Indicative of anti-hnRNP A1 IgG’s biological activity and potential pathogenicity, further studies showed that anti-hnRNP A1 antibodies inhibited neuronal firing using whole-cell current clamp recordings of individual rat neurons (*ex vivo*) and immunostaining immunoreactivity to hnRNP A1 was greatest in the corticospinal system of human brain (Levin et al., 2002a; Kalume et al., 2004).

With a close biological and pathological relationship between HAM/TSP and MS, it was hypothesized that MS patients might also develop autoantibodies to hnRNP A1. Our lab confirmed this in later studies, and further found that the immunodominant epitope of the autoimmune IgG response was specific for the M9 domain (Figure 1B; Lee et al., 2011; Levin et al., 2012, 2013; Douglas et al., 2013). It was also found that anti-hnRNP A1 antibodies entered neuronal cells through clathrin-mediated endocytosis in neuronal cell lines (Mohamed et al., 2002; Douglas et al., 2013) and exacerbated the nucleocytoplasmic mislocalization of hnRNP A1 in animal models of disease (Figure 3; Lee B.J. et al., 2006; Lee et al., 2011; Levin et al., 2012, 2013). Subsequent studies showed that *in vitro* neuronal cell lines exposed to anti-hnRNP A1 monoclonal antibodies, again overlapping the human immunodominant M9 epitope, developed: (Chung et al., 2018) increased markers of neurodegeneration, apoptosis and reduced levels of ATP (Liu et al., 2017) changes in gene expression related to the clinical phenotype of progressive MS patients and (Kashima et al., 2007) mislocalization of hnRNP A1 from the nucleus to the cytoplasm (Douglas et al., 2013, 2016).

Observations in neurons of MS brains have shown the cytoplasmic mislocalization of hnRNP A1. We initially reported hnRNP A1 mislocalization from the nucleus to the cytoplasm, where it co-localized with SGs, in neurons of a brain from an MS patient with aggressive disease (Salapa et al., 2018). In a more recent study, we carried out a comprehensive analysis of over 2700 neurons examined from twelve MS compared to six healthy control brains showed nucleocytoplasmic mislocalization of hnRNP A1 and TDP−43 statistically distinguished MS from control cases (Salapa et al., 2020). There were also neurons in MS cases that displayed nuclear depletion of hnRNP A1 and TDP-43 (Salapa et al., 2020). Nuclear depletion is a marker of neuronal injury and death and was not present in the healthy controls (Salapa et al., 2020). These results were paralleled *in vivo* in mice with experimental autoimmune encephalomyelitis (EAE), an animal model of MS, where hnRNP A1 mislocalization and SG formation are observed in spinal cord neurons of EAE, but not naïve mice (Douglas et al., 2016; Lee et al., 2019; Libner et al., 2020). Additionally, in separate experiments with EAE-induced mice, anti-hnRNP A1 antibodies were injected into EAE mice upon presenting limp tail physiology, a clinical sign of initial disease progression in EAE (Douglas et al., 2016; Libner et al., 2020). In these studies, we observed an exacerbation of clinical phenotypes throughout the EAE time-course, and upon visualization of spinal cord motor neurons at all cord levels, found predominant localization of antibodies to ventral gray matter motor neurons, significant increases in neuronal hnRNP A1 mislocalization and SG formation in thoracic and lumbar regions, and significant neuronal cell loss in thoracic and lumbar motor neurons (Douglas et al., 2016; Libner et al., 2020). Furthermore, these mice also displayed spastic paraparesis, a common clinical feature of the progressive MS phenotype (Douglas et al., 2016; Libner et al., 2020). Throughout these experiments with tissue culture, animal models, and human tissues, we have found a common theme of hnRNP A1 mislocalization and association with induced SGs (Figure 3); other data demonstrates that this can be regulated by several PTMs in response to acute stressors, and so it remains to be seen how this is regulated under chronic inflammatory stress signaling.

While germline mutations in hnRNP A1 have not been described in the pathogenesis of MS, an accumulation of somatic mutations in hnRNP A1 were identified in lymphocytes of progressive MS patients. Specifically, experiments showed novel single nucleotide variants (SNVs) in hnRNP A1, in the PrLD domain (e.g., c.793A > G/N265D) and M9 sequence (e.g., c.841T > C/F281L) (Lee and Levin, 2014). Experimentally, it was shown that transfection of these hnRNP A1 mutants into neuronal-like cells influenced hnRNP A1 cytoplasmic mislocalization, SG formation, and binding to Transportin-1 (Figure 3; Lee and Levin, 2014; Salapa et al., 2018). While the specific mechanisms of how hnRNP A1 mutations contribute to dysfunction are currently unknown, results indicate an influence in altered SG biology, altered RNA metabolism and the induction of apoptosis (Figure 3). Overall, the discovery of somatic mutations in hnRNP A1 in MS supports a tripartite hypothesis, where an environmental trigger, in a genetically susceptible individual, causes an autoimmune response to CNS antigens that results in MS pathology (Lee and Levin, 2014).

As it is still mechanistically unknown how hnRNP A1 dysregulation leads to MS pathogenesis, some targets have been proposed. Recently, a study showed that hnRNP A1 contributes to myelin associated glycoprotein (MAG) RNA splicing by interacting with a motif in intron 12, immediately downstream of the 5′ splice site in exon 12 (Zearfoss et al., 2013). Molecularly, *MAG* mRNA can occur as two alternatively spliced isoforms, a shorter variant that includes exon 12 and contains a termination codon (*S-MAG*), and a longer variant where exon 12 is spliced out (*L-MAG*) (Quarles, 2007). The authors show that hnRNP A1 binding blocks 5′-splice site recognition in *MAG* pre-mRNA, inhibiting the inclusion of exon 12, thereby forming the *L-MAG* mRNA isoform (Zearfoss et al., 2013). They further confirmed this by reducing the expression of hnRNP A1 with siRNA and showed an increase in *MAG* exon 12 inclusion, producing more *S-MAG* isoform mRNA (Zearfoss et al., 2011). While a potential disruption in the S-MAG/L-MAG ratio has yet to be specified in MS, a study using the EAE mouse model observed a depletion in MAG protein levels in the spinal cord, resulting in an increase in PARP-1 cleavage, and an increase in apoptosis activation in glial and neuronal cells (Skundric et al., 2008). This cleavage abrogates the PARylation activity of PARP-1, preventing its ability to regulate hnRNP A1 accumulation in SGs (Blom et al., 2004; Duan et al., 2019). Additionally, antibodies toward MAG have been found in the CSF of MS patients, and MAG is thought to be a specific target of neuroinflammation, leading to its role in the dysregulation of axonal demyelination (Moller et al., 1989; Baig et al., 1991). Overall, these results suggest the control of *MAG* RNA splicing by hnRNP A1 may be an interesting target for further study, as well as present another avenue where hnRNP A1 dysregulation may influence MS pathogenesis.

### Alzheimer’s Disease

Alzheimer’s disease (AD) is characterized by the formation of amyloid-β and Tau aggregates, leading to neuronal dysfunction and death. Current research suggests that hnRNP A1 may influence the formation of both amyloid-β and Tau aggregates. HnRNP A1 is theorized to affect amyloid-β formation through its effect on the alternative splicing of amyloid precursor protein (APP) pre-mRNA (Donev et al., 2007). Specifically, hnRNP A1 has been reported to influence the splice variation of pre-mRNA *APP* through binding to *Alu* RNA elements that are located on either side of exon 7 (Donev et al., 2007). The RNA transcribed from the *Alu* elements in the sense orientation contain a sequence of GCGGA that partially matches the high-affinity binding sequence of the RRM in hnRNP A1 (Donev et al., 2007). By binding to *Alu* elements, hnRNP A1 influences the alternative splicing of both exons 7 and 8, thereby affecting the generation of APP splice variants (Figure 3; Donev et al., 2007). Reduced expression of hnRNP A1, which has been observed in neurons of AD patients, limits the production of *APP* mRNA with skipped exons 7 and 8 (*APP770* mRNA) (Figure 3; Donev et al., 2007). To remedy this, studies suggest treatment with estradiol may result in an increased expression of hnRNP A1, and the formation of spliced *APP* mRNA that produces lower levels of amyloid-β (*APP695* mRNA) (Donev et al., 2007; Villa et al., 2011). Additionally, cholinergic signaling may also play a role in controlling hnRNP A1 expression, and a loss of cholinergic neurons, as observed in AD, may contribute to hnRNP A1 down-regulation, and modified alternative splicing of *APP* mRNA (Berson et al., 2012). Interestingly, the effect of kinase inhibitors to prevent hnRNP A1-mediated alternative splicing on exclusion of exons 7 and 8 from *APP* mRNA has not been explored.

In addition to potentially influencing amyloid-β production, hnRNP A1 has also been reported to affect the splicing of the receptor for advanced glycation end-products (*RAGE*). Amyloid-β binding to membrane-bound RAGE leads to its improper activation, resulting in a chronic cellular immune response and dysfunction and death of neurons (Lue et al., 2001; Son et al., 2012). It has been shown that increased expression of hnRNP A1 is associated with changes in two splice variants of *RAGE*, including an increase in membrane RAGE expression, and a decrease in secretory RAGE expression in AD patients (Nozaki et al., 2007; Liu et al., 2015).

Another study indicates that hnRNP A1 influences AD by modulating *TAU* splicing. The authors show that hnRNP A1 promotes the formation of *3R-TAU* mRNA through the exclusion of exon 10 (Liu et al., 2020). Mechanistically, the authors suggest that hnRNP A1 inhibits the splicing of intron 9, but not intron 10, through a direct interaction with a 3′ splice site in exon 10 of *TAU* pre-mRNA, thus promoting the exclusion of exon 10 (Liu et al., 2020). Reduction in hnRNP A1 expression using siRNA led to an increase in both exon 10 inclusion in *TAU* pre-mRNA, leading to an increase in 4R-Tau protein expression (Liu et al., 2020). This study, however, only demonstrates a correlation between hnRNP A1 dysfunction and tauopathy modulation in AD, and further research is needed to ascertain whether this mechanism affects the pathogenesis of AD. Overall, the effect of phosphorylation or unconventional ubiquitination of hnRNP A1 on alternative splicing of these AD-associated mRNAs have not been characterized, but it is likely to be relevant since this could provide a means to modulate all three of these dysregulated mRNAs.

### Huntington’s Disease

Recently, hnRNP A1 been suggested to influence Huntington’s disease (HD) through mitochondrial mechanisms. Current data shows that hnRNP A1 regulates the expression of dynamin-related protein 1 (Drp1), through an interaction with the 3′-UTR of *DRP1* mRNA (Park et al., 2015). Drp1 is a GTPase protein primarily located in the cytosol and is recruited to the mitochondria where it acts in a concert with other proteins to promote mitochondrial fission using GTPase hydrolysis (Shirendeb et al., 2012). This is important in HD because reports have shown that Drp1 expression is increased in the post-mortem brains of HD patients and data indicate that Drp1 dysfunction, caused by mHtt, may result in excessive mitochondrial fission and fragmentation (Song et al., 2011; Shirendeb et al., 2012; Guo et al., 2013). While the specifics of the hnRNP A1 and *DRP1* mRNA interaction are unknown, or whether hnRNP A1 influences *DRP1* mRNA splicing or stability, the data does show that hnRNP A1 does not significantly change the levels of *DRP1* mRNA, suggesting hnRNP A1 control of Drp1 does not affect the generation of *DRP1* pre-mRNA (Park et al., 2015). Furthermore, current data shows that downregulating hnRNP A1 with siRNA results in a significant decrease in Drp1 protein expression, while the opposite was observed with hnRNP A1 overexpression (Figure 3; Park et al., 2015). This suggests a pro-translational effect of hnRNP A1 on *DRP1*, although no IRES has been identified for *DRP1.* Morphologically, downregulation of hnRNP A1 resulted in increased mitochondrial fusion and decreased fragmentation, both indicators of mitochondrial dysfunction (Park et al., 2015). Additionally, 3-nitropropionic acid (3-NP) treatment, an inhibitor of mitochondrial complex II, paired with hnRNP A1 downregulation, leads to cell death and caspase activation inhibition, and there is subsequent recovery of mitochondrial membrane potential and ATP levels (Park et al., 2015). Based on these results, it is suggested that hnRNP A1 dysregulation, caused possibly by either overexpression or cell stress meditation, potentially causes the enhanced expression of Drp1, leading to mitochondrial dysfunction (Figure 3; Park et al., 2015). The specific mechanism of how this occurs, however, have not been reported, but further investigation of hnRNP A1 in HD would help elucidate its role in pathogenesis.

## Conclusion

While hnRNP A1 has been studied for over 40 years, only recently has hnRNP A1 dysfunction been shown to contribute to the pathogenesis of neurodegenerative disease. The functional view of hnRNP A1 has evolved past its depiction as a protein primarily involved in RNA metabolism, to a protein involved in the specific regulation of many cellular processes that have important functions in health and disease. Research describing such functions as LLPS, membraneless organelle formation, and protein aggregation, have consistently shown that hnRNP A1 contributes significantly to both normal and perturbed function, and may play a greater role than once thought. The mechanisms that hnRNP A1 utilizes to regulate each function, however, are not completely understood, but ongoing and future research aim to clarify and augment current knowledge, with a goal to develop therapeutics. This is especially exemplified in current SMA research, where new knowledge on hnRNP A1 endogenous function has led to successful therapeutic development.

Adding to the complexity of hnRNP A1 in endogenous and perturbed molecular functions are PTMs. While there has been extensive characterization of the effects of myriad PTMs on hnRNP A1 function in cell survival and acute stress responses, research in the context of neurodegenerative disease has been lagging. Although neurodegenerative disease has not extensively been the focus of hnRNP A1 research to date, it is still reasonable to theorize that PTMs may influence pathogenesis and may be an eventual target for disease therapeutics.

As there are still gaps in our understanding of hnRNP A1 regulation and dysfunction in neuronal health, continued research into the mechanisms and outcomes of hnRNP A1 dysfunction will help elucidate the contribution of hnRNP A1 in neurodegenerative disease and hopefully lead to therapies for improved outcomes.

## Author Contributions

JC designed the figures and tables, and was the primary writer of the manuscript. PT contributed to manuscript content and edited the manuscript. HS reviewed and edited the manuscript. ML reviewed and edited the manuscript, and approved the final version, including figures. All authors contributed to the article and approved the submitted version.

## Conflict of Interest

The authors declare that the research was conducted in the absence of any commercial or financial relationships that could be construed as a potential conflict of interest.
